# Dried MoS_2_–Cobalt Alginate Membrane for Rapid Catalytic Degradation of Methylisothiazolinone

**DOI:** 10.3390/gels11110852

**Published:** 2025-10-25

**Authors:** Minglin Wang, Ye Li, Kun Yang, Rui Liu, Mengqi Wang, Kongyin Zhao

**Affiliations:** 1 State Key Laboratory of Advanced Separation Membrane Materials, Tiangong University, Tianjin 300387, China; 2331020438@tiangong.edu.cn (M.W.); 2431020400@tiangong.edu.cn (K.Y.); 2431020373@tiangong.edu.cn (R.L.); 2430020237@tiangong.edu.cn (M.W.); 2 Research Institute for Environmental Innovation (Binhai Tianjin), Tianjin 300457, China

**Keywords:** cobalt alginate, molybdenum disulfide, advanced oxidation technology, drying, catalytic degradation, methylisothiazolinone

## Abstract

The rapid development of industry has led to the discharge of large quantities of organic pollutants into water bodies, posing a significant threat to aquatic safety. It is imperative to develop efficient and environmentally friendly methods for the elimination of organic pollutants. The integration of hydrogel membranes with advanced oxidation processes (AOPs) for water purification has attracted considerable interest due to their high efficiency. However, conventional wet membrane materials stored in aqueous environments are more prone to swelling and leakage of loaded metal species. This limits its application in the degradation of organic pollutants. This study employs a vacuum drying strategy for wet hydrogels, incorporating molybdenum disulfide as a cocatalyst and Co^2+^ cross-linking within the alginate matrix, resulting in a dried MoS_2_–cobalt alginate hydrogel membrane (D-MoS_2_-CoAlg). The drying process of the D-MoS_2_-CoAlg membrane not only significantly enhanced its mechanical strength and anti-swelling capacity but also effectively mitigated the leaching of Co^2+^. Throughout five consecutive cycles, the concentration of leached Co^2+^ remained below 0.032 mg/L. This enables the membrane to achieve a balance between reusability and environmental compatibility. Under the conditions of a drying time of 60 min, a peroxymonosulfate (PMS) dosage of 0.2 mmol/L, and an initial methylisothiazolinone (MIT) concentration of 20 mg/L, the D-MoS_2_-CoAlg membrane exhibited exceptional catalytic performance, achieving a degradation rate of MIT as high as 92.14% within 5 min. The D-MoS_2_-CoAlg membrane demonstrates high catalytic activity and good stability, showing promising potential for application in the field of organic wastewater treatment.

## 1. Introduction

The rapid development of urban industrialization has exacerbated water pollution issues [1]. The widespread use of organic additives such as antibiotics, preservatives, and flame retardants has posed serious threats to water safety [2]. Although antibiotics are indispensable in the medical field, their ability to remain active after entering water bodies means that even trace amounts can endanger human health [3]. Methylisothiazolinone (MIT) effectively inhibits microbial growth and extends product shelf life [4], can still cause allergic reactions due to its low toxicity, and has become one of the major allergens in European countries. Current elimination methods for methylisothiazolinone in aquatic environments have primarily focused on photodegradation. Activating peroxymonosulfate (PMS) via simulated ultraviolet irradiation or direct natural sunlight exposure represents an effective strategy. However, photodegradation suffers from limitations including prolonged treatment duration and the formation of transformation products that retain chronic toxicity [5,6,7]. Therefore, the efficient and environmentally friendly degradation of such organic pollutants in water is of significant research importance [8,9].

Persulfate-based advanced oxidation processes (PS-AOPs) can efficiently degrade refractory organic pollutants in wastewater [10]. The core mechanism involves activating persulfates (e.g., PMS) to generate reactive oxygen species (ROS), primarily sulfate radicals (SO_4_^−^·) and hydroxyl radicals (·OH) [11,12,13]. These highly oxidative radicals effectively attack and break down contaminants, ultimately mineralizing them (e.g., into CO_2_ and H_2_O) or converting them into readily degradable intermediates [14]. Among various activation methods, transition metal activation (e.g., using Co^2+^, Fe^2+^, Cu^2+^, etc.) has attracted significant attention due to its high efficiency [15]. These metals catalytically decompose PMS, enabling sustained radical generation. Notably, in heterogeneous activation pathways, transition metals (or their compounds) can release ions at a controlled rate and regenerate active sites, significantly prolonging the oxidant’s persistence and enabling deep degradation of pollutants [16]. Among these, Co^2+^ exhibits particularly outstanding activation performance and holds the greatest application potential [17]. Single-atom catalysts refer to supported catalysts in which individual metal atoms are dispersed on a carrier surface, serving as active sites [18]. The use of single-atom catalysts for PMS activation offers maximized atom utilization efficiency and enhances the catalytic performance per unit mass of metal [19]. Despite their high catalytic activity and role in advancing AOPs, they cannot entirely circumvent the limitations inherent in heterogeneous catalytic systems [20]. Emerging issues such as high preparation costs, rapid deactivation, and difficult recovery of catalysts have become prominent challenges. The development of novel activators characterized by high efficiency, stability, low cost, and environmental friendliness serves as an effective approach to address the aforementioned challenges.

Single-atom catalysts supported on hydrogel interfaces have been extensively studied for their high efficiency [21]. Alginate hydrogel membranes offer a high metal loading capacity due to their abundant functional groups. Moreover, the raw material is derived from natural algae, making it environmentally friendly and cost-effective [22]. Using hydrogel membranes as supports to anchor metal atoms overcomes the difficulties associated with conventional single-atom catalyst preparation [23], such as complex synthesis and limited loading capacity. Chen et al. [24] investigated and compared the catalytic performance of alginate hydrogel membranes anchoring different metal ions through a one-step cross-linking approach. This method enables ultrafast degradation of organic pollutants within confined interfaces. However, it suffers from limited reusability. When pollutant-containing solutions permeate through the hydrogel membrane under a pressure-driven field, significant membrane swelling occurs in the continuous reaction environment [25], which reduces the service life. The loaded metal atoms may undergo reduction or oxidation, leading to changes in their valence states, and often leach from the support into the reaction solution, resulting in metal loss [26]. This constitutes an inevitable issue when using conventional wet hydrogel membranes for catalytic degradation.

Therefore, we first introduced MoS_2_ as a co-catalyst, followed by drying the conventional wet hydrogel membrane to effectively address the aforementioned issues. The incorporation of MoS_2_ primarily functions as a support matrix to coordinate with Co, thereby optimizing the activation efficiency of PMS. The introduction of MoS_2_ as a cocatalyst during the preparation process has been extensively studied [27] and represents a feasible strategy to effectively inhibit metal ion leaching. Li et al. [28] synthesized magnetic CoFe_2_O_4_ nanoparticle/MoS_2_ catalysts, in which MoS_2_ provides reduction sites for Co^3+^/Fe^3+^ during the reaction and forms a type-II heterojunction with CoFe_2_O_4_, thereby enhancing catalytic capability. By incorporating MoS_2_, this system enables direct activation of PMS through electron transfer on the one hand, and on the other hand, MoS_2_ acts as a robust support for Co, facilitating the rapid redox cycling of the Co^3+^/Co^3+^ pair and maintaining catalytic activity throughout the reaction [29]. The introduction of nanomaterials brings significant benefits to catalyst regeneration and reusability [30]. Drying-mediated dehydration of hydrogel membranes can significantly enhance their anti-swelling capacity and tensile stress. Xu et al. substantially improved the mechanical strength of carboxylated multiwalled carbon nanotubes/calcium alginate (CMWCNT/CaAlg) membranes through vacuum drying, thereby enhancing their operational stability [31].

Herein, we innovatively propose a strategy involving Co^2+^ cross-linked sodium alginate (NaAlg) hydrogel matrix incorporated with MoS_2_ as a co-catalyst. Through further vacuum drying treatment, we successfully overcame the limitations of conventional wet hydrogel membranes and fabricated a D-MoS_2_-CoAlg dried hydrogel membrane. This membrane possesses high-density Co atomic sites at the reaction interface, which efficiently activate PMS, demonstrating exceptional degradation efficiency and cycling performance in the degradation of organic pollutant MIT. Additionally, the resulting membrane exhibits significant advantages in mechanical properties and anti-swelling capacity. This work provides novel insights for the elimination of organic pollutants in wastewater.

## 2. Results and Discussion

### 2.1. Surface Morphology Analysis of D-MoS_2_-CoAlg Hydrogel Membrane

**Figure 1:**
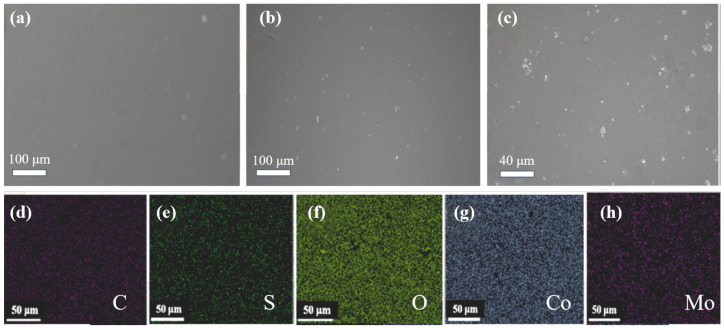
SEM image of the CoAlg membrane (**a**), SEM images of D-MoS_2_-CoAlg membranes at different magnifications (**b**,**c**), and EDS spectra of D-MoS_2_-CoAlg membranes (**d**–**h**).

Figure 1a shows a Scanning electron microscope (SEM) image of a CoAlg membrane without MoS_2_ loading, revealing a smooth, flat, and densely structured surface. Figure 1b,c present SEM images of the D-MoS_2_-CoAlg membrane at different magnifications. Both at high and low magnifications, the membrane appears flat and dense without visible porous structures. Uniformly dispersed MoS_2_ particles can be clearly observed, adhering closely to the membrane matrix.

Figure 1d–h show the Energy dispersive spectrometer (EDS) spectra of the D-MoS_2_-CoAlg hydrogel catalytic membrane, illustrating the distribution of five elements—C, O, Mo, S, and Co—within the membrane. All elements are uniformly distributed. Table 1 also lists the weight percentage contents of these elements in the membrane. Among them, Co exhibits the highest content at 45.16 wt.%, while Mo accounts for 1.60 wt.%, confirming the successful doping of MoS_2_.

### 2.2. FTIR and XRD Analysis of D-MoS_2_-CoAlg Hydrogel Membrane

**Figure 2:**
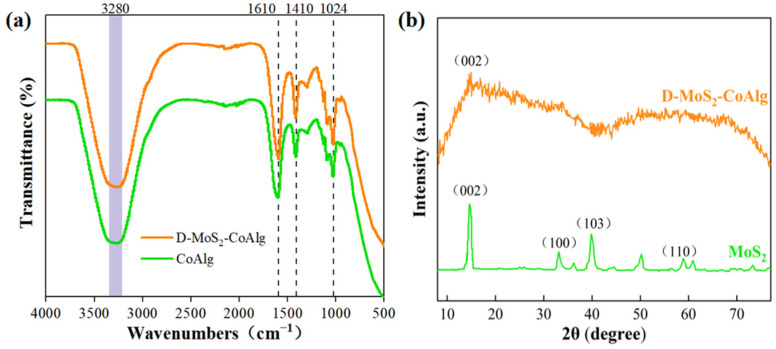
FT-IR spectra (**a**) of D-MoS_2_-CoAlg hydrogel membrane and CoAlg membrane, XRD patterns of D-MoS_2_-CoAlg hydrogel membrane and MoS_2_ (**b**).

Figure 2a shows the Fourier transform infrared spectrometer (FT-IR) spectra of the D-MoS_2_-CoAlg hydrogel membrane and the pure CoAlg membrane. As illustrated, the infrared curve trends of the D-MoS_2_-CoAlg hydrogel membrane and the pure CoAlg membrane are generally consistent, indicating that the introduction of MoS_2_ did not significantly alter the main functional group structure of CoAlg. The peak at 1024 cm^−1^ corresponds to the stretching vibration of C–OH, the peak at 1410 cm^−1^ is attributed to the symmetric stretching vibration of –COO^−^, and the peak at 1610 cm^−1^ is assigned to the asymmetric stretching vibration of –COOH. The broad absorption peak around 3280 cm^−1^ represents the stretching vibration of –OH [32]. These characteristic peaks confirm the presence of an alginate-based cross-linked membrane structure. At approximately 3280 cm^−1^ and 1024 cm^−1^, the absorption intensity of D-MoS_2_-CoAlg is slightly enhanced, which may be related to interactions such as hydrogen bonding or coordination between MoS_2_ and sodium alginate [33].

Figure 2b displays the X-ray diffraction (XRD) patterns of MoS_2_ and the D-MoS_2_-CoAlg hydrogel catalytic membrane. As shown, four diffraction peaks are observed in the MoS_2_ pattern at 2θ values of 14.7°, 32.9°, 39.8°, and 58.9°, corresponding to the (002), (100), (103), and (110) crystal planes, respectively [34]. The positions and intensities of these peaks match the standard card data (PDF # 09-0077). Meanwhile, a sharp diffraction peak at the (002) crystal plane is clearly visible in the pattern of the D-MoS_2_-CoAlg membrane, confirming the successful incorporation of MoS_2_ into the composite membrane. In the pattern of the D-MoS_2_-CoAlg membrane, the peak intensity is significantly reduced [35]. This can be attributed to the relatively low loading of MoS_2_ and its encapsulation within the alginate gel matrix, which suppresses its crystalline diffraction.

### 2.3. Analysis of the Mechanical Properties and Swelling Resistance of D-MoS_2_-CoAlg Hydrogel Membrane

**Figure 3:**
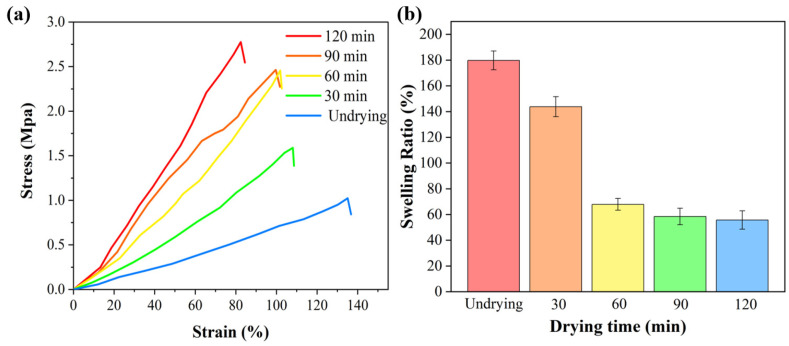
Mechanical properties (**a**) and swelling properties (**b**) of D-MoS_2_–CoAlg membranes.

Figure 3a shows the stress–strain curves of D-MoS_2_-CoAlg hydrogel membranes subjected to different drying times. Drying duration significantly influenced the mechanical properties of the membranes: with prolonged drying time, the fracture strength gradually increased, while the elongation at break gradually decreased. Longer drying times resulted in a notable improvement in membrane strength but a reduction in toughness. This is attributed to the loss of water molecule plasticization and the associated toughening mechanism. During the drying process of the hydrogel, interchain interactions are significantly enhanced, leading to restricted molecular mobility and consequently reduced toughness [36].

Figure 3b illustrates the changes in the anti-swelling performance of the composite membranes under different drying times. To minimize significant experimental errors caused by variations in membrane sample quality, the maximum swelling ratio in the anti-swelling experiments of the D-MoS_2_-CoAlg membrane was determined by calculating the average value from multiple membrane samples. The membrane without drying exhibited poor anti-swelling ability, with a swelling ratio reaching 179.78% at 120 min. Moderate drying significantly enhanced the anti-swelling performance of the D-MoS_2_-CoAlg hydrogel membrane. As indicated in the figure, the membrane dried for 60 min demonstrated excellent anti-swelling properties, with a swelling ratio of only 67.89% at 120 min. For hydrogel membranes, controlled drying promotes the formation of more stable physical cross-linking points between polymer chains within the membrane, thereby increasing the robustness and density of the network [37]. Prolonged drying time resulted in increased brittleness of the membrane, while the anti-swelling capacity remains almost unchanged. Based on a comprehensive consideration of the membrane’s tensile strength, toughness, and anti-swelling capacity, the membrane with a drying duration of 60 min was selected for subsequent catalytic experiments.

### 2.4. Effect of Different Drying Durations on MIT Removal Efficiency by D-MoS_2_-CoAlg Membrane

**Figure 4:**
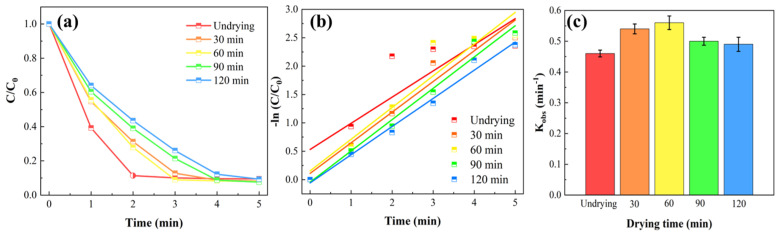
MIT removal efficiency by D-MoS_2_-CoAlg membrane under different drying times (**a**), pseudo-first-order kinetic fitting curves (**b**), and reaction rate constants (**c**).

As shown in Figure 4a, the drying time significantly influenced the catalytic performance of the membranes. Similar to the trends observed in mechanical and anti-swelling properties, the membrane dried for 60 min exhibited the optimal catalytic efficiency. The undried membrane (0 min drying) achieved an MIT (20 ppm) removal rate of 90.52% within 5 min. After drying, the degradation efficiency of MIT improved under the same duration, with the removal rate increasing to 92.14% at 5 min. Furthermore, as calculated and shown in Figure 4c, the reaction rate constant of the membrane dried for 60 min reached 0.56 min^−1^, accompanied by the highest MIT removal efficiency. When the drying time exceeds 60 min, the polymer chains are forced into close proximity, forming strong physical interactions. Upon rehydration of the dried membrane in an aqueous environment, the polymer network cannot fully restore its pre-dried expanded state [38]. While Co^2+^ ions bind to specific coordination sites on the polymer chains, some remain physically encapsulated within the aqueous phase. Due to excessive drying, some Co^2+^ ions cannot rapidly interact with PMS under the pressure-driven field to generate free radicals as efficiently as before. This delayed encounter consequently slows the degradation rate of MIT, thereby reducing the corresponding reaction rate constant.

### 2.5. Effect of PMS Concentration on MIT Removal Efficiency

**Figure 5:**
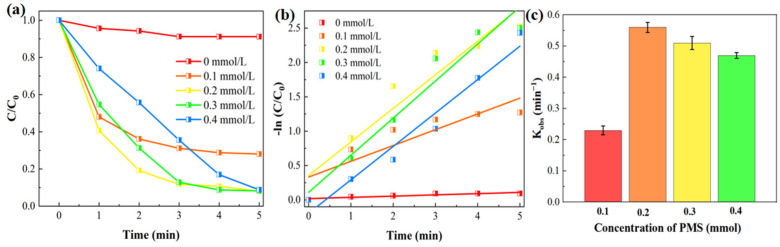
MIT removal efficiency at different PMS concentrations (**a**), pseudo-first-order kinetic fitting curves (**b**), and reaction rate constants (**c**).

The concentration of PMS is a critical factor influencing the removal efficiency of MIT. During the catalytic reaction process, the single-atom catalysts loaded within the membrane undergo electron exchange with PMS [39]. The Co species and MoS_2_ in the D-MoS_2_–CoAlg membrane serve as electron transfer mediators and provide active sites, thereby inducing redox reactions in PMS to generate strongly oxidizing species, including sulfate radicals (SO_4_^−^·), hydroxyl radicals (·OH), and singlet oxygen (^1^O_2_) [40]. Thus, efficient elimination of MIT can be achieved. This study investigated the MIT (20 ppm) removal performance of the D-MoS_2_-CoAlg membrane at different PMS concentrations. As shown in Figure 5, at a PMS concentration of 0.1 mM, the MIT removal rate reached 70.42% within 5 min. When the concentration was increased to 0.2 mM, the removal rate rose to 92.14% at approximately 5 min. Further increasing the PMS concentration to 0.3 mM resulted in a removal rate of 90.80% at around 5 min, while at 0.4 mM, the removal rate slightly decreased to 90.52%. This phenomenon occurs because a fixed amount of PMS generates a limited quantity of reactive species [41]. At lower concentrations, the amount of reactive species produced is insufficient to completely degrade the pollutants. In contrast, excessively high concentrations may lead to radical self-quenching reactions, which reduce catalytic performance and increase operational costs [42]. Therefore, the optimal PMS concentration was determined to be 0.2 mM.

### 2.6. Effect of MIT Initial Concentration on Its Removal Efficiency

**Figure 6:**
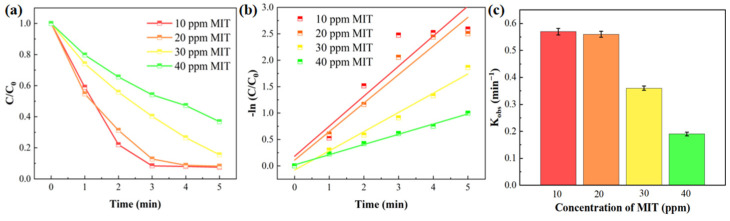
Effect of initial MIT concentration on the removal efficiency of D-MoS_2_-CoAlg membranes (**a**), pseudo-first-order kinetic fitting curves (**b**), and reaction rate constants (**c**).

The initial concentration of organic pollutants also directly affects their removal efficiency. Figure 6a shows the removal efficiency of MIT at different initial concentrations by the D-MoS_2_-CoAlg membrane. The results indicate that the removal efficiency of MIT decreases as its initial concentration increases. This occurs because, at lower initial concentrations of MIT, the amount of reactive species generated from PMS is sufficient to degrade the pollutants. As the initial concentration of MIT increases while the PMS concentration remains unchanged, the total amount of reactive species produced remains constant, leading to incomplete degradation of the pollutants [7] and thus a decrease in removal efficiency. Based on the fitted curves and rate constants in Figure 6b,c, the degradation rates at MIT concentrations of 10 ppm and 20 ppm are similar. The reaction rate constant at these concentrations is 2.95 times higher than that at 40 ppm. An MIT concentration of 20 ppm is also more representative of real wastewater conditions. Therefore, it can be concluded that the D-MoS_2_-CoAlg membrane exhibits high efficiency in degrading organic pollutants.

### 2.7. Active Species Analysis

**Figure 7:**
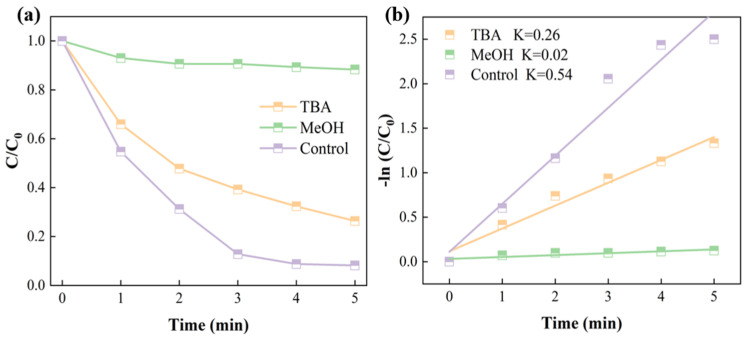
Quenching experiment of active species (**a**); dynamics fitting curve of quenching experiment (**b**).

The types of active species present in the catalytic system were investigated by conducting quenching experiments through the addition of quenchers to the D-MoS_2_-CoAlg membrane catalytic system. As shown in Figure 7, after adding 24.30 mL of methanol (MeOH), the MIT (20 ppm) removal rate decreased significantly, reaching only 10.31% at 5 min, indicating the presence of ·OH in the system. In contrast, after adding 57.38 mL of tert-butanol (TBA), the MIT removal rate slightly decreased to 73.24% at 5 min, suggesting the existence of SO_4_^−^·in the catalytic system.

**Figure 8:**
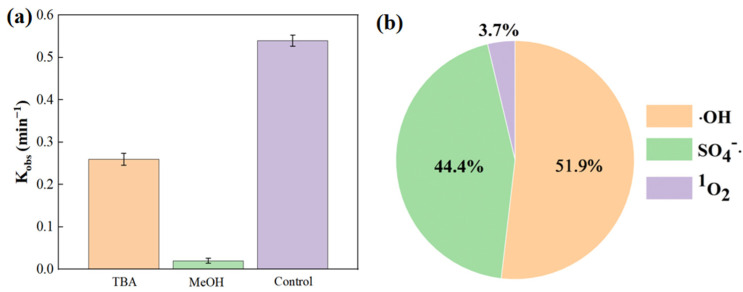
Reaction rate constant of quenching experiment (**a**); contributions of SO_4_^−^·, ·OH, and ^1^O_2_ in catalytic system (**b**).

To further clarify the roles of various active species, the reaction rate constants were obtained by kinetic fitting of the quenching experimental results, enabling the calculation of the contribution of each active species to the catalytic reaction. The resulting reaction rate constants from the quenching experiments are presented in Figure 8a. The results show that after adding TBA, the k value was 0.26 min^−1^, while after adding MeOH, the k value was only 0.02 min^−1^. Using Equations (4)–(6), the contributions of different active species in the catalytic system were calculated. As shown in Figure 8b, ·OH contributed the most in the catalytic system, reaching 51.85%, followed by SO_4_^−^·at 44.44%, while the contribution of ^1^O_2_ was only 3.71%. This demonstrates that the active species in the system are predominantly radicals, with ·OH being the primary active species. Additionally, the non-radical active species ^1^O_2_ is present but plays a minor role.

### 2.8. Reusability of D-MoS_2_-CoAlg Hydrogel Membrane

**Figure 9:**
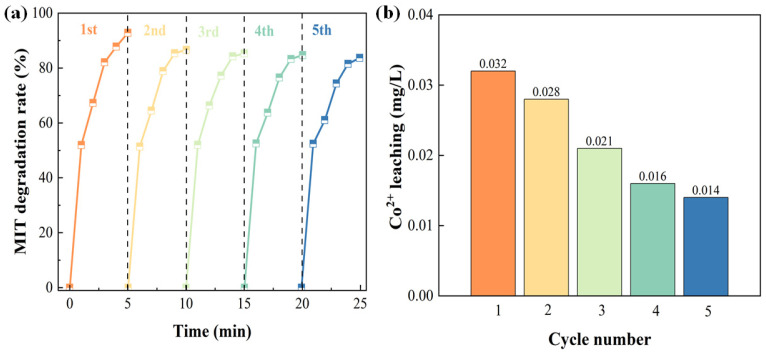
Reusability of the D-MoS_2_-CoAlg hydrogel membrane (**a**); Co^2+^ leaching concentration after cycle test (**b**).

Reusability tests were conducted on the D-MoS_2_-CoAlg hydrogel membrane for five consecutive cycles, and the results are presented in Figure 9a. Within five minutes, the MIT (20 ppm) removal rates were 92.14%, 86.70%, 85.30%, 84.70%, and 83.66%, respectively. These results demonstrate that the hydrogel membrane possesses excellent reusability, along with high durability and stability.

Typically, moist hydrogel membranes possess a porous structure, which allows metal ions to leach out under a pressure-driven field [43]. The vacuum drying process induces acute contraction of the hydrogel network, leading to closely packed polymer chains [44]. This structural change hinders the leakage of metal ions through diffusion pathways. When the dried D-MoS_2_-CoAlg hydrogel membrane is reintroduced to an aqueous environment, molecules from the solution must first penetrate and swell the network to reopen the channels before any encapsulated metal ions can diffuse out. This process significantly delays the leaching rate of metal ions. The incorporation of MoS_2_ also plays a positive role in enhancing the membrane’s reusability. As a cocatalyst, MoS_2_ forms a synergistic catalytic system with Co^2+^, and its ability to anchor metal ions further reduces ion leakage [45]. The enhanced role of MoS_2_ as a cocatalyst in the D-MoS_2_-CoAlg system can be attributed to three key aspects. First, the Mo sites provide a significant reducing capability. Second, the unsaturated S atoms generate abundant S vacancies, which positively influence the PMS activation process [46]. Finally, there exists a pairing interaction between the transition metal Co and MoS_2_. The lone pair electrons possessed by S atoms may form coordination bonds with Co^2+^, while the Mo atoms at the edge sites of MoS_2_ nanosheets with unsaturated coordination are readily substituted by Co atoms [47]. These synergistic interactions accelerate the regeneration and evolution between different valence states of Co. Figure 9b presents the concentration of Co^2+^ leaching from the D-MoS_2_-CoAlg membrane during cyclic experiments, as detected by ICP analysis. The results indicate that the leaching concentration of Co^2+^ remained below or equal to 0.032 mg/L throughout all five cycles. Consequently, the D-MoS_2_-CoAlg hydrogel membrane demonstrates excellent reusability. Moreover, limiting the leakage of Co^2+^ aligns with the principles of sustainable development by preventing potential adverse environmental impacts.

### 2.9. Performance Comparison of D-MoS_2_-CoAlg Membrane with Other Catalytic Membranes

Table 2 shows the performance comparison between D-MoS_2_-CoAlg membrane and other catalytic membranes in the literature. We conducted a comprehensive comparison from three aspects, PMS concentration, pollutant concentration, and catalytic efficiency. Using the reaction rate constant as the evaluation criterion for catalytic performance. According to the table data, the D-MoS_2_-CoAlg membrane has significant performance advantages and exhibits excellent catalytic efficiency at extremely low PMS concentrations (0.2 mmol/L). The reaction rate reached 0.56 min^-1^. This has significant economic advantages in efficiently degrading MIT (20 ppm).

## 3. Conclusions

This study successfully prepared a dried MoS_2_–CoAlg (D-MoS_2_–CoAlg) composite catalytic membrane based on sodium alginate and molybdenum disulfide. The mechanical strength and anti-swelling properties of D-MoS_2_–CoAlg membrane were significantly enhanced through vacuum drying treatment. The D-MoS_2_–CoAlg membrane exhibited excellent catalytic activity in a persulfate-based advanced oxidation process (PS-AOPs), achieving a 92.14% degradation rate of MIT (20 ppm) within 5 min under optimal conditions (drying time: 60 min; PMS concentration: 0.2 mmol/L). Quenching experiments confirmed that hydroxyl radicals (·OH) played a dominant role in the degradation process. Furthermore, the membrane exhibits good reusability, maintaining a degradation efficiency of over 83% after five consecutive cycles, with minimal leaching of Co^2+^. The drying process and the anchoring effect between MoS_2_ and Co^2+^ further reduce ion leaching. Overall, the D-MoS_2_–CoAlg membrane demonstrates considerable practical potential for organic wastewater treatment. In the future, we will continue to explore the functions of different two-dimensional materials (such as MXene, g-C_3_N_4_, etc.) as co-catalysts to advance research on catalytic activity and stability. Evaluate the environmental impact of membrane materials throughout their entire lifecycle and promote their green and sustainable development.

## 4. Materials and Methods

### 4.1. Materials

Sodium alginate (NaAlg, food grade) was procured from Qingdao Mingyue Algae Group Co., Ltd. (Qingdao, China) Hexahydrate cobalt chloride (CoCl_2_·6H_2_O, AR), methanol (MeOH, AR), tert-butanol (TBA, AR), molybdenum disulfide (MoS_2_, AR), potassium persulphate (PMS, AR), 2-Methyl-4-isothiazolin-3-one (MIT, AR) were all purchased from Shanghai Aladdin Reagent Co., Ltd. (Shanghai, China).

### 4.2. Preparation of D-MoS_2_-CoAlg Hydrogel Catalytic Membrane

**Figure 10:**
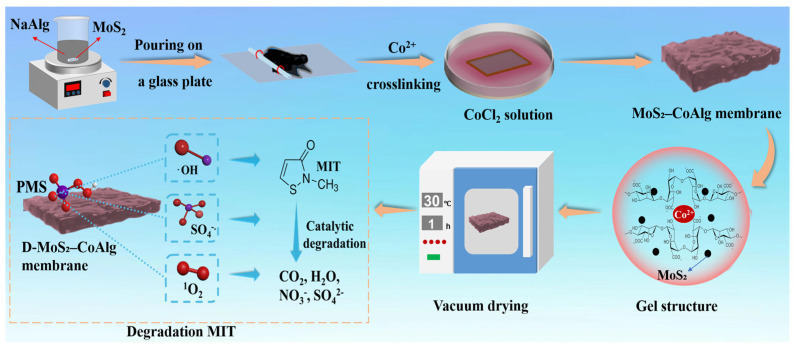
Schematic illustration of the preparation and application of D-MoS_2_-CoAlg membrane.

Figure 10 illustrates the schematic diagram of the preparation process and application of the D-MoS_2_-CoAlg membrane. A certain volume of MoS_2_ dispersion (mass percentage concentration of 1 wt.%) was added into a beaker and uniformly mixed with deionized water. Then, 2.5 wt.% NaAlg was slowly added, and the mixture was mechanically stirred for 8 h using a magnetic stirrer. After stirring, the beaker was removed and allowed to stand for 12 h for deaeration. Subsequently, a small amount of the casting solution was poured onto a flat, dry glass plate and cast using a copper wire (diameter 0.5 mm). The glass plate coated with the casting solution was immersed in a 1 wt.% CoCl_2_ solution (the molar concentration is 0.077 mol/L) for cross-linking to obtain the MoS_2_-CoAlg membrane. The cross-linking time was 8 h. After cross-linking, the MoS_2_-CoAlg membrane was taken out and rinsed with deionized water to remove residual metal ions on the surface. It was then placed in a vacuum oven for drying (drying time: 0, 30, 60, 90, and 120 min; drying temperature: 30 °C) to obtain D-MoS_2_-CoAlg membranes with different degrees of dryness. The thickness of the membrane exhibited marked variation with differing drying times. As drying time increased, the membrane thickness decreased. The thickness of the undried membrane was 0.217 mm. The thicknesses of the membranes dried for 30 min, 60 min, 90 min, and 120 min were 0.190 mm, 0.157 mm, 0.121 mm, and 0.039 mm, respectively.

### 4.3. Characterization of D-MoS_2_-CoAlg Membrane

The surface morphology of the D-MoS_2_-CoAlg membranes was characterized using a digital camera and a thermal field emission scanning electron microscope (TFE-SEM, Model B110G500, ZEISS, Oberkochen, Germany). The elemental distribution and content on the membrane surface were analyzed by an energy dispersive spectrometer (EDS, Model Ultim Max 65, Oxford, UK). The structure and functional groups of the MoS_2_-CoAlg hydrogel catalytic membranes were examined using a Fourier transform infrared spectrometer (FT-IR, Model Nicolet iS50, Thermo Scientific, Waltham, MA, USA). The crystal structure and material composition of the membranes were determined via X-ray diffraction (XRD, Model D8 DISCOVER, Bruker, Bremen, Germany) under the following conditions: maximum tube voltage: 60 kV; maximum tube current: 80 mA; scanning angle range (2θ): 10–80°; scanning speed: 5°/min. The leaching concentration of Co^2+^ in the cyclic catalytic experiments was detected using inductively coupled plasma mass spectrometry (ICP MS 7700X, Agilent Technologies, Santa Clara, CA, USA).

### 4.4. Mechanical Properties Testing

The mechanical properties of the D-MoS_2_-CoAlg hydrogel catalytic membranes were measured using a single-fiber strength tester (Model LLY-06F, Laizhou Electronic Instrument Co., Ltd., Laizhou, China). First, the membranes were cut into approximately uniform strips (typically 10 mm × 5 mm). The thickness of each membrane was measured at five different points using a micrometer thickness gauge, and the average value was calculated. Tensile tests were then performed on the membranes using the strength tester, with ten repetitions conducted for each type of membrane and the results averaged. The testing parameters were as follows: room temperature; initial grip separation: 10 mm; tensile rate: 10 mm/min; break threshold: 80%; linear density: 1 dtex. The elongation at break (λ) was calculated according to Equation (1):

(1) $$\lambda \;=\;(L\;-\;L_{0})/L_{0}\;\;\times \;100$$

In the above equation, *L*_0_ represents the length of the composite membrane before stretching (mm), and *L* denotes the length of the composite membrane after stretching (mm).

### 4.5. Catalytic Degradation Performance Testing

A laboratory-assembled cross-flow filtration setup was employed to investigate the catalytic degradation performance of the D-MoS_2_-CoAlg hydrogel catalytic membrane toward the MIT. The membrane surface was rinsed with deionized water to remove residual metal ions, after which the membrane was placed in the filtration cell (effective filtration area: 19.6 cm^2^). A certain concentration of MIT solution was added to the feed tank. The system was pre-pressurized with deionized water at 0.1 MPa for 30 min, followed by the addition of a specific concentration of PMS to initiate the catalytic degradation experiment. At designated time intervals, samples were collected from the feed tank, and their absorbance was measured using a UV–Vis spectrophotometer to record the experimental results. Among them, the MIT wavelength is 273 nm. The removal rate R of the pollutant was calculated according to Equation (2). Furthermore, the elimination of the pollutant followed pseudo-first-order kinetics, and the reaction rate constant k was determined using Equation (3).

(2) $$R=(C_{0}-C)/C_{0}\times 100\%$$

(3) $$-ln(C/C_{0})=kt$$

In the aforementioned equation, *C*_0_ denotes the initial concentration of the pollutant (mg/L), *C* represents the concentration of the pollutant at a given time (mg/L), *k* is the reaction rate constant (min^−1^), and *t* is the reaction time (min).

### 4.6. Quenching Experiment of Active Species

Quenching experiments using different quenchers were conducted to investigate the active species present in the reaction system and their respective roles. Methanol (MeOH, 3 M) was used as a quencher for both SO_4_^−^· and ·OH, while tert-butanol (TBA, 3 M) served as a quencher specific to ·OH. In the feed tank, 200 mL of MIT with a concentration of 20 mg/L was added, and the system was pre-pressurized at 0.1 MPa for 30 min. Then, 24.30 mL of methanol and 57.38 mL of tert-butanol were introduced separately, followed by the addition of 0.2 mmol/L PMS to initiate the quenching experiments. The contribution rates of each active species in the catalytic system were calculated according to Equations (4)–(6).

(4) $$\lambda \left[\cdot \mathrm{OH}\right]=(k_{0}-k_{1})/k_{0}\times 100\%$$

(5) $$\lambda \left[{{\mathrm{SO}}_{4}}^{-}\cdot \right]=\left(k_{1}-k_{2}\right)/k_{0}\times 100\%$$

(6) λO21=100−λ·OH−λSO4−·

In these equations, λ[OH], λ[SO_4_^−^·], and λ[^1^O_2_] represent the contributions of ·OH, SO_4_^−^·, and ^1^O_2_, respectively. k_0_ denotes the reaction rate constant without the addition of any quencher, k_1_ represents the reaction rate constant of the system when TBA is added, and k_2_ indicates the reaction rate constant of the system when MeOH is added.

## Figures and Tables

**Table 1:** Surface element content of D-MoS_2_-CoAlg hydrogel membrane.

Membrane	Element	Content (wt.%)
D-MoS_2_-CoAlg	C	18.96
	O	33.21
	Co	45.16
	Mo	1.60
	S	1.07

**Table 2:** Comparison of the performance of different catalysts for the removal of organic pollutants.

Catalyst	Oxidant (mM)	Pollutant (mg/L)	Removal Efficiency	k_obs_ (min^−1^)	Ref.
Fe_1_/CN	PMS (1)	BPA (22.8)	100% (10 min)	1.43	[48]
Co-N_3_O_1_	PMS (1)	CIP (5)	100% (20 min)	0.287	[49]
Co-N_2 + 2_-C	PMS (0.98)	CIP (20)	100% (40 min)	0.1403	[50]
SACo-NGs	PMS (1)	BPA (22.8)	100% (5 min)	0.6	[51]
Fe-SACs	PMS (1.3)	BPA (25)	88% (30 min)	0.104	[52]
Mn-ISAs@CN	PMS (0.65)	BPA (20)	100% (4 min)	1.138	[53]
SA-Fe-NC	PMS (2)	BPA (22.8)	100% (3 min)	1.99	[54]
Cu-N_4_/C-B	PMS (0.65)	BPA (20)	98% (5 min)	0.56	[55]
CoAlg	PMS (0.1)	MIT (30)	100% (1 min)	4.0	[24]
D-MoS_2_-CoAlg	PMS (0.2)	MIT (20)	92.14% (5 min)	0.56	This work

## Data Availability

The original contributions presented in this study are included in the article. Further inquiries can be directed to the corresponding authors.

## Abbreviations

·OH	Hydroxyl radicals
CaAlg	Calcium alginate
CMWCNT	Carboxylated multiwalled carbon nanotubes
D-MoS_2_-CoAlg	Dried MoS_2_-cobalt alginate
EDS	Energy dispersive spectrometry
FT-IR	Fourier transform infrared spectrometry
ICP-MS	Inductively coupled plasma mass spectrometry
MIT	Methylisothiazolinone
MoS_2_	Molybdenum disulfide
MoS_2_-CoAlg	MoS_2_-cobalt alginate
NaAlg	Sodium alginate
PMS	Peroxymonosulfate
PS-AOPs	Persulfate-based advanced oxidation processes
ROS	Reactive oxygen species
SO_4_^−^·	Primarily sulfate radicals
TFE-SEM	Thermal field emission scanning electron microscope
XRD	X-ray diffraction
